# Classification, risk factors, and outcomes of patients with progressive hemorrhagic injury after traumatic brain injury

**DOI:** 10.1186/s12883-023-03112-x

**Published:** 2023-02-13

**Authors:** Ren Wang, Dian-Xu Yang, Jun Ding, Yan Guo, Wan-Hai Ding, Heng-Li Tian, Fang Yuan

**Affiliations:** Department of Neurosurgery, Shanghai Sixth People’s Hospital Affiliated to Shanghai Jiao Tong University School of Medicine, Shanghai Jiao Tong University, Shanghai, China

**Keywords:** Traumatic brain injury, Progressive hemorrhagic injury, Classification, propensity score matching, outcomes

## Abstract

**Background:**

According to the pathoanatomic classification system, progressive hemorrhagic injury (PHI) can be categorized into progressive intraparenchymal contusion or hematoma (pIPCH), epidural hematoma (pEDH), subdural hematoma (pSDH), and traumatic subarachnoid hemorrhage (ptSAH). The clinical features of each type differ greatly. The objective of this study was to determine the predictors, clinical management, and outcomes of PHI according to this classification.

**Methods:**

Multivariate logistic regression analysis was used to identify independent risk factors for PHI and each subgroup. Patients with IPCH or EDH were selected for subgroup propensity score matching (PSM) to exclude confounding factors before evaluating the association of hematoma progression with the outcomes by classification.

**Results:**

In the present cohort of 419 patients, 123 (29.4%) demonstrated PHI by serial CT scan. Of them, progressive ICPH (58.5%) was the most common type, followed by pEDH (28.5%), pSDH (9.8%), and ptSAH (3.2%). Old age (≥ 60 years), lower motor Glasgow Coma Scale score, larger primary lesion volume, and higher level of D-dimer were independent risk factors related to PHI. These factors were also independent predictors for pIPCH, but not for pEDH. The time to first CT scan and presence of skull linear fracture were robust risk factors for pEDH. After PSM, the 6-month mortality and unfavorable survival rates were significantly higher in the pIPCH group than the non-pIPCH group (24.2% vs. 1.8% and 12.1% vs. 7.3%, respectively, *p* < 0.001), but not significantly different between the pEDH group and the non-pEDH group.

**Conclusions:**

Understanding the specific patterns of PHI according to its classification can help early recognition and suggest targeted prevention or treatment strategies to improve patients’ neurological outcomes.

**Supplementary Information:**

The online version contains supplementary material available at 10.1186/s12883-023-03112-x.

## Background

Progressive hemorrhagic injury (PHI) occurs in up to 60% [1–3] of patients with traumatic brain injury (TBI). It is a devastating but potentially modifiable risk factor for the worse outcomes after TBI. We previously developed and validated a prediction score to identify those patients at highest risk of PHI [4]. However, the heterogeneity of PHI is a significant barrier to selecting appropriate risk factors to predict the different subtypes of PHI and to evaluate the effects of early therapeutic intervention for PHI on outcomes after TBI [5].

To identify specific risk factors associated with the subtype of PHI remains one of the most significant challenges in PHI prediction and management. Pathoanatomic classification is used most often to describe the anatomical features of injuries for acute management in TBI [6]. According to the pathoanatomic classification system [7], traumatic intracerebral hemorrhage can be categorized into intraparenchymal contusion or hematoma (IPCH), epidural hematoma (EDH), subdural hematoma (SDH), and traumatic subarachnoid hemorrhage (tSAH). PHI subtypes including those IPCH, EDH, SDH, and tSAH that progress. Classification of PHI could be used to link specific patterns of PHI with corresponding predictors and evaluate the effects of a target intervention on outcomes [6]. It is worth evaluating how a therapy could effectively treat a particular subtype of PHI. This will also help physicians to choose accurate risk factors to identify the occurrence of a PHI subtype and decide an effective treatment strategy to improve outcomes or their prediction after primary TBI.

PHI prediction and outcome determination varies in the literature, partially caused by enrollment of different type of PHI [2, 7–10]. In this preliminary study, we investigated risk factors and their influence on treatment strategy and association with outcomes of PHI by pathoanatomic classification.

## Methods

### Patient population and characteristics

We reviewed all cases of adults TBI treated at Shanghai Sixth People’s Hospital, People’s Republic of China during a 2-year period (January 2016 to December 2018) using the hospital’s electronic medical record system. Similar to our previous inclusion criteria [4], patients who were diagnosed as isolated TBI with at least 2 CT scans were included. We excluded (1) patients underwent surgical intervention after the first CT scan, although the follow up CT after surgery was available; (2) patients with known coagulation disorders; and (3) patients with intracranial pathological changes before their injury. Accordingly, of the initial 576 consecutive TBI patients, 419 patients remained for subsequent analysis.

Demographic and clinical variables were collected as follows: age, gender, mechanism of injury, Glasgow Coma Scale (GCS) score, motor GCS score, pupil reactivity, time to the first computed tomography (CT) scan, skull fracture, primary lesion volume, EDH, tSAH, intraventricular hemorrhage (IVH), midline shift, cistern compression, D-dimer, length of hospital stay (LOS), posttraumatic cerebral hydrocephalus, posttraumatic cerebral infarction, and surgical interventions including hematoma evacuation and decompressive craniectomy (DC).

All enrolled patients were dichotomized into PHI (those IPCH, EDH, SDH, and tSAH that progress) and non-PHI groups (those IPCH, EDH, SDH, and tSAH that did not progress). Within the PHI group, patients were further divided into progressive IPCH, EDH, SDH, and tSAH subgroups. For patients with TBI exhibit mixed picture of hemorrhage, the pathoanatomic type of PHI was recorded as the major proportion of hematoma/contusion. Because the events of pSDH and ptSAH were infrequent, only patients with IPCH or EDH were selected for subgroup propensity score matching (PSM) [11].

Neurological outcome was recorded using the 6-month score on the Glasgow Outcome Scale (GOS). The 6-month GOS was split into dead (score = 1), unfavorable survival (2 or 3), and favorable survival (4 or 5). All data were collected by regular outpatient follow-up or telephone interview.

### CT scan image analysis

All patients received first CT scan at admission. For those patients with hemorrhage on the admission CT without clinical deterioration, a second CT scan was performed routinely at about 6 h after presentation; for those patients with clinical deterioration, a second CT scan was performed immediately. The time of PHI occurrence was record as the evolving on the second CT scan both routine and immediate repeat CT scan following clinical deterioration. A proficient neuroradiologist who was blind to the patients’ characteristics evaluated all CT images. From the CT scan, types of intracranial hemorrhage were recorded as IPCH, EDH, SDH, or tSAH. A midline shift was defined as ≥ 5 mm. The areas of the brain contusion or hematoma were measured using ImageJ software (National Institutes of Health, Bethesda, MD, USA) on each 5-mm CT scan slice; lesion volume was then calculated by multiplying the sum of all areas by the interval distance. The occurrence of PHI was confirmed by both the neuroradiologist and neurosurgeons using the previous PHI definition, which was defined as the appearance of new lesions or a conspicuous increase in the size of hemorrhagic lesions (i.e., a 25% increase or more compared to the first post-injury CT scan; Oertel et al., 2002; Sanus et al., 2004) [4, 7, 10].

### Statistical analysis

Categorical variables were expressed as percentages or constituent ratio. Pearson’s chi-square test was used to evaluate group differences. The Fisher’s exact test was used when one or more of the cell counts in an R × C table is less than 5. Continuous variables were expressed as mean ± standard deviation. One way analysis of variance was used for variables that fulfilled the criteria of normal distribution and equal variance; otherwise the Kruskal–Wallis test was used. A stepwise strategy was used in logistic regression analysis for PHI. The variables included in the initial regression models were listed in the supplemental Tables 1, 2, and 3.

The propensity score for each patient was calculated using the patients’ baseline demographics, injury severity, and CT scan characteristics. Each patient who developed PHI was matched to the patient in the non-PHI group who had the closest propensity score with a simple 1:2 nearest-neighbor matching algorithm. To exclude bad matches, we imposed a caliper of 0.02 of the standard deviation of the logit of the propensity score. All *p*-values < 0.05 were considered significant. Statistical analyses were performed with SPSS 24.0 (SPSS Inc., Chicago, IL, USA) and R for Windows version 3.2.3 (The R Foundation for Statistical Computing, Vienna, Austria) software.

## Results

### Pathoanatomic classification of PHI

Of the 419 patients, 123 (29.4%) demonstrated PHI by serial CT scan. PHI was categorized into progressive IPCH, EDH, SDH, and tSAH by the pathoanatomic classification system. Of the 123 patients with PHI, 72 (58.5%) developed progressive IPCH, 35 (28.5%) developed progressive EDH, 12 (9.8%) developed progressive SDH, and four (3.2%) developed progressive tSAH. Progressive IPCH appeared after injury within the first 6 h in 11 (15.3%), 6 − 12 h in 25 (34.7%), 12 − 24 h in 27 (37.5%), and after 24 h in nine (12.5%) patients. Progressive EDH appeared after injury within the first 6 h in 21 (60%), 6 − 12 h in six (17.1%), and 12 − 24 h in eight (22.9%) patients. Progressive SDH appeared after injury within the first 6 h in three (25%), 6 − 12 h in six (50%), 12 − 24 h in two (16.7), and after 24 h in one (8.3%) patient. Progressive tSAH appeared after injury within 6 − 12 h in one (25%), 12 − 24 h in one (25%), and after 24 h in two (50%; Table 1) patients.

**Table 1:** Pathoanatomic classification of progressive hemorrhagic injury (PHI), showing the time after initial injury that it developed

PHI type	0 − 6 h	6 − 12 h	12 − 24 h	24 − 48 h	48 − 72 h	72 − 96 h	Total
pIPCH	11 (15.3)	25 (34.7)	27 (37.5)	7 (9.7)	1 (1.4)	1 (1.4)	72 (100)
pEDH	21 (60.0)	6 (17.1)	8 (22.9)	0 (0.0)	0 (0.0)	0 (0.0)	35 (100)
pSDH	3 (25.0)	6 (50.0)	2 (16.7)	1 (8.3)	0 (0.0)	0 (0.0)	12 (100)
ptSAH	0 (0.0)	1 (25.0)	1 (25.0)	0 (0.0)	2 (50.0)	0 (0.0)	4 (100)
Total	35 (28.5)	38 (30.9)	38 (30.9)	8 (6.5)	3 (2.4)	1 (0.8)	123 (100)

*pEDH* progressive epidural hematoma, *pIPCH* progressive intraparenchymal contusion or hematoma, *pSDH* progressive subdural hematoma, *ptSAH* progressive traumatic subarachnoid hemorrhage

### Risk factors of PHI by pathoanatomic classification

The patient characteristics are listed in Table 2 by PHI pathoanatomic classification. In general, univariate analysis revealed that older age, lower GCS, poorer pupil reactivity, larger primary lesion volume, higher serum level of D-dimer, and appearance of EDH, tSAH/IVH, midline shift, and cistern compression are associated with PHI. Patients with PHI had a significantly higher risk of worse outcomes and longer LOS than patients who did not develop PHI. In the PHI subgroup analysis, the patients with progressive EDH were younger, with larger primary lesion volume, lower serum level of D-dimer, shorter LOS, and better outcomes compared to the patients with other types of PHI.

**Table 2:** Patient characteristics in the progressive hemorrhagic injury (PHI) subgroups and non-PHI group

Characteristics	Category	Non-PHI n (%)	PHI n (%)			*P*- value
			pIPCH	pEDH	pSDH/tSAH	
Age	Mean ± SD	47.19 ± 19.91	57.94 ± 14.36	40.17 ± 13.12	46.31 ± 20.38	.000*
	≥ 60 years	52 (17.6)	34 (47.2)	2 (5.7)	4 (25.0)	.000^†^
Gender	Male	219 (74.0)	60 (83.3)	29 (82.9)	12 (75.0)	.296^§^
Mechanism	Motor vehicle	152(51.4)	40(55.6)	22(62.9)	10(62.5)	
	Fall	77(26.0)	15(20.8)	8(22.9)	5(14.3)	
	Assault	15(5.1)	3(4.2)	5(14.2)	0(0)	
	Other	52(17.5)	14(19.4)	0(0)	1(6.2)	.038^†^
GCS score	3 − 8	34 (11.5)	25 (34.7)	13 (37.1)	12 (75.0)	
	9 − 12	38 (12.8)	21 (29.2)	7 (20.0)	0 (0.0)	
	13 − 15	224 (75.7)	26 (36.1)	15 (42.9)	4 (25.0)	.000^†^
Motor score	1 − 2	10 (3.4)	2 (2.8)	0 (0.0)	7 (43.8)	
	3	8 (2.7)	4 (5.6)	2 (5.7)	2 (12.5)	
	4	14 (4.7)	15 (20.8)	7 (20.0)	1 (6.3)	
	5	45 (15.2)	27 (37.5)	12 (34.3)	2 (12.5)	
	6	219 (74.0)	24 (33.3)	14 (40.0)	4 (25.0)	.000^†^
Pupil	Both reactive	274 (92.6)	63 (87.5)	29 (82.9)	8 (50.0)	
	one reactive	8 (2.7)	3 (4.2)	4 (11.4)	3 (18.8)	
	Non-reactive	14 (4.7)	6 (8.3)	2 (5.7)	5 (31.3)	.000^†^
Time to 1^st^ CT scan (h)	< 3 h	58	19	11	3	
	3–6 h	144	33	18	10	
	> 6 h	94	20	6	3	.349^†^
Fracture	Linear	148	47	34	8	.000^†^
Primary lesion volume	Mean ± SD	13.32 ± 21.93	24.42 ± 23.06	44.46 ± 45.14	19.93 ± 31.32	.003^#^
EDH	Yes	82 (27.7)	8 (11.1)	30 (85.7)	2 (12.5)	.000^†^
tSAH/IVH	Yes	167 (56.4)	46 (63.9)	12 (34.3)	13 (81.3)	.006^†^
Midline shift	≥ 5 mm	27 (9.1)	7 (9.7)	10 (28.6)	4 (25.0)	.004^†^
Cistern compression	normal	248 (83.8)	51 (70.8)	15 (42.9)	11 (68.8)	
	compressed	36 (12.2)	14 (19.4)	16 (45.7)	1 (6.3)	
	absent	12 (4.1)	7 (9.7)	4 (11.4)	4 (25)	.000^†^
D-dimer (mg/L)	Mean ± SD	7.22 ± 13.74	17.25 ± 21.69	11.20 ± 16.02	33.54 ± 30.31	0.000^#^
6-month outcome	Dead	7 (2.4)	19 (26.4)	3 (8.6)	7 (43.8)	
	Unfavorable survival	12 (4.1)	15 (20.8)	3 (8.6)	4 (25.0)	
	Favorable survival	277 (93.6)	38 (52.8)	29 (82.9)	5 (31.3)	.000^†^
LOS	Days	19.2 ± 18.55	32.1 ± 32.27	21.80 ± 14.69	40.44 ± 64.01	0.00^#^
PTCH	Yes	5 (1.7)	6 (8.3)	0 (0.0)	3 (18.8)	.001^†^
PTCI	Yes	4 (1.4)	4 (5.6)	0 (0.0)	1 (6.3)	.057^†^

*CT* computed tomography, *pEDH* progressive epidural hematoma, *GCS* Glasgow Coma Scale, *pIPCH* progressive intraparenchymal contusion or hematoma, *IVH* intraventricular hemorrhage, *LOS* length of hospital stay, *PTCH* posttraumatic cerebral hydrocephalus, *PTCI* posttraumatic cerebral infarction, *SD*: standard deviation, *pSDH* progressive subdural hematoma, *ptSAH* progressive traumatic subarachnoid hemorrhage

^*^one way ANOV analysis

^†^Fisher’s exact test

^§^Chi-squared test

^#^Kruskal–Wallis test

Multivariate logistic regression revealed that old age (≥ 60 years), lower motor GCS score, larger primary lesion volume, and higher level of D-dimer are independent risk factors related to PHI (Table 3). PHI subgroup analysis found that these factors are also independently associated with progressive IPCH (Table 4), but are not associated with progressive EDH. The time to first CT scan and a skull linear fracture are robust risk factors for progressive EDH (Table 5).

**Table 3:** Multivariate logistic analysis of risk factors associated with progressive hemorrhagic injury after traumatic brain injury^a^

Risk factor	Odds ratio	95% confidence interval	*P*-value
Age ≥ 60 years	2.10	1.24 − 3.58	.006
Motor score	1.41	1.11 − 1.79	.005
Primary lesion volume	1.01	1.00 − 1.02	.010
D-dimer (mg/L)	1.02	1.01 − 1.03	.004

^a^*N* = 419, stepwise logistic regression

**Table 4:** Multivariate logistic analysis of risk factors associated with progressive intraparenchymal contusion or hematoma^a^

Risk factor	Odds ratio	95% confidence interval	*P*-value
Age ≥ 60 years	2.48	1.34 − 4.60	.004
Motor score	1.40	1.05 − 1.86	.022
Primary lesion volume	1.03	1.01 − 1.04	.001
D-dimer (mg/L)	1.02	1.00 − 1.03	.024

^a^N = 297, stepwise logistic regression

**Table 5:** Multivariate logistic analysis of risk factors associated with progressive epidural hematoma^a^

Risk factors	Odds ratio	95% confidence interval	*P*-value
Time to 1^st^ computed tomography scan	2.10	1.24 − 3.58	.006
Fracture	1.41	1.11 − 1.79	.005

^a^*N* = 122, stepwise logistic regression

### Management of PHI: repeated CT scans, influences of intervention and surgical management

The mean time between the first and second CT scans was 6.9 ± 3.6 h. The volume of progressive IPCH on the second CT scan was 16.96 ± 17.31 mL with a range of 1 − 81.5 mL. Forty-nine of the 72 patients who developed progressive IPCH received surgical intervention, and 44 of them underwent DC. The volume of progressive EDH on the second CT scan was 26.41 ± 22.66 mL with a range from 3.7 − 83.7 mL. Twenty-seven of the 35 patients who developed progressive EDH received hematoma evacuation, and five of them underwent DC. The volume of progressive SDH on the second CT scan was 26.22 ± 22.36 mL with a range of 6.7 − 75 mL. Ten of the 12 patients who developed progressive SDH received surgical intervention, and all of 10 patients underwent DC. The volume of progressive tSAH was not calculated and this did not significantly influence the treatment strategy (Table 6).

**Table 6:** Surgical intervention in progressive hemorrhagic injury (PHI)

Type of PHI	Volume (mean ± standard deviation, mL)	Volume (range, mL)	No. of surgical interventions n (%)	No. of DCs n (%)
pIPCH	16.96 ± 17.31	1 − 81.5	49 (68.1)	44 (61.1)
pEDH	26.41 ± 22.66	3.7 − 83.7	27 (77.1)	5 (14.3)
pSDH	26.22 ± 22.36	6.7 − 75	10 (83.3)	10 (83.3)
ptSAH	-	-	0	0
Total	20.20 ± 19.52	1 − 83.7	86	59

*DC* decompressive craniectomy, *pEDH* progressive epidural hematoma, *pIPCH* progressive intraparenchymal contusion or hematoma, *pSDH* progressive subdural hematoma, *ptSAH* progressive traumatic subarachnoid hemorrhage

### Subtype of PHI and outcomes

In the unadjusted data, the PHI group had a significantly worse outcome rate than the non-PHI group. The 6-month mortalities were 2.4% in the non-PHI group, and 26.4% in the progressive IPCH, 8.6% in the progressive EDH, and 43.8% in the progressive SDH/tSAH subgroups. The 6-month unfavorable survival rates were 4.1%, 20.8%, 8.6%, and 25%, respectively (Table 2). To minimize the influence of confounding variables on the accuracy of outcome assessment, we used the patients’ baseline demographics, injury severity, and CT scan characteristics to calculate the values for PSM. We did not find a significant difference in the variables between the PHI and non-PHI groups under this analysis (Supplemental Table 4). The PHI group still had significantly higher 6-month mortality and unfavorable survival than the non-PHI group (20.0% vs. 3.2% and 10.0% vs. 7.3%, respectively, *p* < 0.001, Table 7). Under the PHI stratified analyses, the 6-month mortality and unfavorable survival were also significant higher in the pIPCH group than the non-pIPCH group (24.2% vs. 1.8% and 12.1% vs. 7.3%, respectively, *p* < 0.001, Table 7). However, the 6-month mortality and unfavorable survival were not significantly different between the pEDH group and the non-pEDH group (0% vs. 4.0% and 0% vs. 12.0%, respectively, *p* = 0.264, Table 7).

**Table 7:** Subtypes of progressive hemorrhagic injury (PHI) and their outcomes after propensity score matching (PSM)

Characteristics	Category	PHI *N* = 80 n (%)	Non-PHI *N* = 124 n (%)	*p*-value	pIPCH *N* = 33 n (%)	Non-pIPCH *N* = 55 n (%)	*p*-value	pEDH *N* = 15 n (%)	Non-pEDH *N* = 25 n (%)	*P*-value
6-month outcome	Dead	16 (20.0)	4 (3.2)		8 (24.2)	1 (1.8)		0 (0.0)	1 (4.0)	
	Unfavorable survival	8 (10.0)	9 (7.3)		4 (12.1)	4 (7.3)		0 (0.0)	3 (12.0)	
	Favorable survival	56 (70.0)	111 (89.5)	.000^*^	21 (63.6)	50 (90.9)	.001^*^	15 (100.0)	21 (84.0)	.372^*^
LOS		23.60 ± 25.44	22.67 ± 21.89	.781^†^	27.36 ± 22.22	22.69 ± 22.18	.342^†^	20.27 ± 9.03	21.40 ± 23.00	.857^†^
PTCH	Yes	3 (3.8)	3 (2.4)	.681^*^	1 (3.0)	2 (3.6)	-^*^	0	1 (4.0)	-^*^
PTCI	Yes	3 (3.8)	3 (2.4)	.681^*^	0	0	-	0	1 (4.0)	-^*^

*pEDH* progressive epidural hematoma, *pIPCH* progressive intraparenchymal contusion or hematoma, *LOS* length of hospital stay, *PTCH* posttraumatic cerebral hydrocephalus, *PTCI* posttraumatic cerebral infarction, *SD* standard deviation

^*^Fisher Exact test

^†^t-test

## Discussion

With the derivation of a risk score for PHI prediction [4] and adoption of a modified CT repeat strategy in our department [12], identification of PHI became more timely and early clinical intervention was given more promptly. PHI can be categorized into progressive IPCH, EDH, SDH, and tSAH by the pathoanatomic classification system. The clinical features of each type of PHI are highly variable. The heterogeneity of PHI compounds the difficulty in its prognosis after primary TBI. The present study dedicated to analyze clinical characteristics and their association with outcomes of PHI by pathoanatomic classification.

The enrollment criteria for PHI varies in the literature. PHI prediction and outcome determination were hampered by a lack of standardized PHI definition and classification. Some studies include progressive ICPH, EDH, SDH, and tSAH [2, 7–10, 13], while others include only IPCH and SDH [14], IPCH [5, 15–17], or EDH [18]. Some studies did not clarify the type of PHI enrolled [2, 8]. Moreover, none of the studies classifying PHI improved understanding of its disparate patterns. In the present study, we reveal that the general rate of PHI after TBI is 29.4% and that progressive ICPH (58.5%) is the most common type of PHI, followed by progressive EDH (28.5%), SDH (9.8%), and tSAH (3.2%).

The clear difference in the incidence of the subtypes of PHI motivated us to link specific patterns of PHI with corresponding risk factors. Multivariate logistic regression demonstrated that old age (≥ 60 years), lower GCS motor score, larger primary lesion volume, and higher level of D-dimer are independent risk factors for PHI. These factors are also independent predictors for progressive IPCH, but are not significantly associated with progressive EDH. The different factors between progressive IPCH and EDH reflect the different mechanisms of these forms of PHI. In patients with IPCH, older age, injury severity, and coagulopathy contribute to its progression. This indicates that early medical intervention to treat TBI-associated coagulopathy [8] and preserve the integrity of the neurovascular unit may be key strategies to prevent progressive IPCH [19]. In patients with EDH, time to first CT scan and skull linear fracture are independent predictors. This indicates that early detection and surgical intervention to stop bleeding of the meningeal artery, due to a fractured skull, are the linchpin for treating progressive EDH. Progressive IPCH is the most common type of PHI, which may explain why the risk factors for PHI predict progressive IPCH well but fail to predict progressive EDH. Thus, to improve the accuracy of PHI prediction, specific risk score systems should be developed for each type of PHI in further studies.

Analyzing the clinical features of PHI according to its classification helps to improve its management and outcomes. In general, traumatic intracranial hemorrhage (90.3%) mainly progresses in the first 24 h. Of its types, EDH (60%) is most likely to enlarge in the first 6 h but seldom does so after 24 h; these patients always have rapid, severe, neurobehavioral deterioration and require surgical intervention. Thus, an aggressive CT repeat strategy should be employed in EDH to detect its progression, especially in the early stage after the primary TBI. IPCH did not evolve as fast as EDH in the first 6 h, but still predominately progressed in the first 24 h after initial injury. Thus, we recommend the CT repeat strategy used in our center: an immediate CT scan when a patient’s consciousness declines rapidly or a routine second scan 6 h after the first one, followed by another at 24 h after injury. With the early prediction and detection of PHI by classification, immediate and appropriate treatment can be given. However, whether the early medical intervention of PHI could improve the outcomes or which treatment is effective in which types of PHI has not been determined [4].

The occurrence of PHI changes treatment strategies and affects prognosis [20]. In the present cohort, most patients who developed PHI needed immediate surgical intervention. The rate of DC in patients with progressive IPCH (61.1%) or SDH (83.3%) was significantly higher than in those with progressive EDH (14.28%). Although aggressive treatment was given, patients with PHI still had worse outcomes than patients who did not. However, the difference in the patients’ characteristics between the PHI and non-PHI groups significantly affected the prognostic accuracy of PHI on outcomes after TBI. To get a more accurate estimate of the effect of PHI on the outcomes, PSM was used to exclude confounding factors [21]. We found that the 6-month mortality and unfavorable survival were significantly higher in the progressive IPCH group than in the non-IPCH group (24.2% vs. 1.8% and 12.1% vs. 7.3%, respectively, *p* < 0.001), but not significantly different between the progressive EDH group and the non-EDH group. This indicates that early detection of PHI followed by prompt medical intervention are effective in improving outcomes in patients with progressive EDH but ineffective in those with progressive IPCH. This subset of patients with progressive IPCH should be selected for future clinical studies to look for more effective prevention and treatment strategies to improve their outcomes. Unfortunately, due to the small number of patients with progressive SDH or tSAH, PSM was not performed on them to evaluate any effect on their outcomes.

### Limitations

This preliminary study has several limitations. First, the number of patients with progressive SDH or tSAH in this cohort was small and thus failed to reach sufficient statistical power to identify the independent risk factors relating to hematoma expansion and to evaluate the effects of their progression on outcomes. Second, we did not analyze hematoma enlargement after DC; another pattern of PHI with specific characteristics and outcomes [22]. Finally, there was a selection bias of patients with two sequential CT scans, which may affect the detection of PHI and assessment of outcomes. For example, patients presented large volume of IPCH, EDH, or SDH on admission were more likely to progress in the natural course of TBI than patients who presented with small lesion volume, but most of them underwent emergency surgical intervention before a routine secondary follow up CT was available. According to our exclusion criteria, this selection bias may decrease the occurrence of PHI.

## Conclusions

The clinical characteristics, management, and outcomes differ significantly among progressive IPCH, EDH, SDH, and tSAH. A specific prediction model must be developed for each type of PHI to improve the accuracy of prediction. Early detection of hematoma expansion followed by prompt medical intervention significantly improved outcomes in patients with progressive EDH but not in those with progressive IPCH. Understanding the specific patterns of PHI according to its classification can help early recognition of hematoma expansion and suggest targeted prevention or treatment strategies to improve patients’ neurological outcomes.

## Supplementary Information

**Additional file 1: Supplemental table 1.** Multivariate logistic analysis of risk factors associated with progressive hemorrhagic injury after traumatic brain injury*. **Supplemental table 2.** Multivariate logistic analysis of risk factors associated with progressive intraparenchymal contusion or hematoma*. **Supplemental table 3.** Multivariate logistic analysis of risk factors associated with progressive epidural hematoma*. **Supplemental table 4.** Subtypes of progressive hemorrhagic injury (PHI) and their outcomes after propensity score matching (PSM).

## Data Availability

The raw data supporting the conclusions of this article will be made available by the corresponding author Fang Yuan, without undue reservation.

## Abbreviations

PHI: Progressive hemorrhagic injury
TBI: Traumatic brain injury
pEDH: Progressive epidural hematoma
pIPCH: Progressive intraparenchymal contusion or hematoma
pSDH: Progressive subdural hematoma
ptSAH: Progressive traumatic subarachnoid hemorrhage
GCS: Glasgow Coma Scale

## References

[1] Cepeda S, Gomez PA, Castano-Leon AM, Martinez-Perez R, Munarriz PM, Lagares A (2015). Traumatic intracerebral hemorrhage: risk factors associated with progression. J Neurotrauma.

[2] Wan X, Fan T, Wang S, Zhang S, Liu S, Yang H (2017). Progressive hemorrhagic injury in patients with traumatic intracerebral hemorrhage: characteristics, risk factors and impact on management. Acta Neurochir.

[3] Yuan Q, Sun YR, Wu X, Yu J, Li ZQ, Du ZY (2016). Coagulopathy in traumatic brain injury and its correlation with progressive hemorrhagic injury: a systematic review and meta-analysis. J Neurotrauma.

[4] Yuan F, Ding J, Chen H, Guo Y, Wang G, Gao WW (2012). Predicting progressive hemorrhagic injury after traumatic brain injury: derivation and validation of a risk score based on admission characteristics. J Neurotrauma.

[5] Allison RZ, Nakagawa K, Hayashi M, Donovan DJ, Koenig MA (2017). Derivation of a predictive score for hemorrhagic progression of cerebral contusions in moderate and severe traumatic brain injury. Neurocrit Care.

[6] Saatman KE, Duhaime AC, Bullock R, Maas AI, Valadka A, Manley GT (2008). Classification of traumatic brain injury for targeted therapies. J Neurotrauma.

[7] Oertel M, Kelly DF, McArthur D, Boscardin WJ, Glenn TC, Lee JH (2002). Progressive hemorrhage after head trauma: predictors and consequences of the evolving injury. J Neurosurg.

[8] Folkerson LE, Sloan D, Cotton BA, Holcomb JB, Tomasek JS, Wade CE (2015). Predicting progressive hemorrhagic injury from isolated traumatic brain injury and coagulation. Surgery.

[9] Chieregato A, Fainardi E, Morselli-Labate AM, Antonelli V, Compagnone C, Targa L (2005). Factors associated with neurological outcome and lesion progression in traumatic subarachnoid hemorrhage patients. Neurosurgery.

[10] Sanus GZ, Tanriverdi T, Alver I, Aydin S, Uzan M (2004). Evolving traumatic brain lesions: predictors and results of ninety-eight head-injured patients. Neurosurg Q.

[11] Fu P, Yuan Q, Lv K, Hu J (2020). First Intracranial Pressure Monitoring or First Operation: Which One Is Better?. World Neurosurg.

[12] Ding J, Yuan F, Guo Y, Chen SW, Gao WW, Wang G (2012). A prospective clinical study of routine repeat computed tomography (CT) after traumatic brain injury (TBI). Brain Inj.

[13] Tian HL, Chen H, Wu BS, Cao HL, Xu T, Hu J (2010). D-dimer as a predictor of progressive hemorrhagic injury in patients with traumatic brain injury: analysis of 194 cases. Neurosurg Rev.

[14] Juratli TA, Zang B, Litz RJ, Sitoci KH, Aschenbrenner U, Gottschlich B (2014). Early hemorrhagic progression of traumatic brain contusions: frequency, correlation with coagulation disorders, and patient outcome: a prospective study. J Neurotrauma.

[15] Chang EF, Meeker M, Holland MC (2006). Acute traumatic intraparenchymal hemorrhage: risk factors for progression in the early post-injury period. Neurosurgery.

[16] Narayan RK, Maas AI, Servadei F, Skolnick BE, Tillinger MN, Marshall LF (2008). Progression of traumatic intracerebral hemorrhage: a prospective observational study. J Neurotrauma.

[17] White CL, Griffith S, Caron JL (2009). Early progression of traumatic cerebral contusions: characterization and risk factors. J Trauma.

[18] Chen H, Guo Y, Chen SW, Wang G, Cao HL, Chen J (2012). Progressive epidural hematoma in patients with head trauma: incidence, outcome, and risk factors. Emerg Med Int.

[19] Kurland D, Hong C, Aarabi B, Gerzanich V, Simard JM (2012). Hemorrhagic progression of a contusion after traumatic brain injury: a review. J Neurotrauma.

[20] Qureshi AI, Malik AA, Adil MM, Defillo A, Sherr GT, Suri MF (2015). Hematoma enlargement among patients with traumatic brain injury: analysis of a prospective multicenter clinical trial. J Vasc Interv Neurol.

[21] Yuan Q, Wu X, Cheng H, Yang C, Wang Y, Wang E (2016). Is Intracranial pressure monitoring of patients with diffuse traumatic brain injury valuable? An observational multicenter study. Neurosurgery..

[22] Flint AC, Manley GT, Gean AD, Hemphill JC, Rosenthal G (2008). Post-operative expansion of hemorrhagic contusions after unilateral decompressive hemicraniectomy in severe traumatic brain injury. J Neurotrauma.

