# Supplementary material for: Classification, risk factors, and outcomes of patients with progressive hemorrhagic injury after traumatic brain injury
Source: BMC Neurol. 2023 Feb 13;23:68. doi: 10.1186/s12883-023-03112-x (PMC9926699; doi:10.1186/s12883-023-03112-x)
Supplement: Supplementary file 1 — Additional file 1: Supplemental table 1. Multivariate logistic analysis of risk factors associated with progressive hemorrhagic injury after traumatic brain injury*. Supplemental table 2. Multivariate logistic analysis of risk factors associated with progressive intraparenchymal contusion or hematoma*. Supplemental table 3. Multivariate logistic analysis of risk factors associated with progressive epidural hematoma*. Supplemental table 4. Subtypes of progressive hemorrhagic injury (PHI) and their outcomes after propensity score matching (PSM). [file 12883_2023_3112_MOESM1_ESM.pdf]

Supplemental tables.

Supplemental table 1. Multivariate logistic analysis of risk factors associated with progressive hemorrhagic injury after traumatic brain injury*.

Risk factor	Odds ratio	95% confidence interval	p-value
Age ≥ 60 years	2.20	1.21−4.00	.009
Time to 1st CT scan (h)	1.27	0.90−1.81	.180
Motor score	1.48	1.13−1.93	.005
EDH	1.23	0.69−2.20	.484
Midline shift	0.37	0.14−1.02	.055
Cistern compression	0.80	0.59−2.00	.798
Primary lesion volume	1.02	1.00−1.03	.020
D-dimer (mg/L)	1.02	1.01−1.04	.003

*N=419, initial stepwise logistic regression model.

Supplemental table 2. Multivariate logistic analysis of risk factors associated with progressive intraparenchymal contusion or hematoma*.

Risk factor	Odds ratio	95% confidence interval	p-value
Age ≥ 60 years	2.26	1.20−4.27	.012
Time to 1st CT scan (h)	1.04	0.68−1.58	.872
Motor score	1.53	1.12−2.10	.007
Midline shift	0.43	0.12−1.58	.205
Cistern compression	0.66	0.32−1.38	.272
Primary lesion volume	1.03	1.01−1.05	.002
D-dimer (mg/L)	1.02	1.01−1.05	.001

*N=297, initial stepwise logistic regression model

Supplemental table 3. Multivariate logistic analysis of risk factors associated with progressive epidural hematoma*.

Risk factors	Odds ratio	95% confidence interval	p-value
Age ≥ 60 years	0.39	0.08−1.83	.233
Time to 1st computed tomography scan	1.93	1.07−3.47	.028
Motor score	0.99	0.67−1.47	.956
Midline shift	1.83	0.65−5.16	.253
Fracture	3.28	1.86−5.78	.000
D-dimer (mg/L)	1.00	0.98−1.03	.576

*N=122 , initial stepwise logistic regression model

Supplemental table able 4. Subtypes of progressive hemorrhagic injury (PHI) and their outcomes after propensity score matching (PSM).

Characteristics	Category	PHI N = 80 n (%)	Non-PHI N = 124 n (%)	p-value	IPCH N = 33 n (%)	Non-IPCH N = 55 n (%)	p-value	EDH N = 15 n (%)	Non-EDH N = 25 n (%)	p-value
Age	Mean ± SD	48.49 ± 18.06	48.52 ± 15.31	.988†	53.00 ± 16.35	52.78 ± 14.26	.948†	38.67 ± 13.52	42.92 ± 13.16	.334†
Motor score	1	3 (3.8)	3 (2.4)		0 (0.0)	1 (1.8)		0 (0.0)	1 (4.0)	
	2	3 (3.8)	5 (4.0)		0 (0.0)	1 (1.8)		0 (0.0)	1 (4.0)	
	3	2 (2.5)	4 (3.2)		3 (9.1)	1 (1.8)		1 (6.7)	1 (4.0)	
	4	9 (11.3)	8 (6.5)		4 (12.1)	3 (5.5)		0 (0.0)	0 (0.0)	
	5	23 (28.8)	24 (19.4)		10 (30.3)	11 (20.0)		7 (46.7)	7 (28.0)	
	6	40 (50.0)	80 (64.5)	.361*	16 (48.5)	38 (69.1)	.161*	7 (46.7)	15 (60.0)	.736*
Pupil	Both reactive	67 (83.8)	110 (88.7)		28 (84.8)	50 (90.9)		13 (86.7)	22 (88.0)	

	one reactive	5 (6.3)	5 (4.0)		1 (3.0)	2 (3.6)		2 (13.3)	2 (8.0)	
	Non-reactive	8 (10.0)	9 (7.3)	.587§	4 (12.1)	3 (5.5)	.652*	0 (0.0)	1 (4.0)	.752*
EDH	Yes	27 (33.8)	36 (29.0)	.476§	-	-	-	15	25	-
Lesion volume	Mean ± SD	22.91 ± 24.40	17.93 ± 26.97	.183†	14.60 ± 15.30	9.81 ± 15.37	.159†	26.01 ± 36.61	22.73 ± 30.21	.760†
Midline shift	≥5 mm	13 (16.3)	14 (11.3)	.307§	4 (12.1)	2 (3.6)	.138*	2 (13.3)	4 (16)	.600*
Cistern compression	normal	56 (70.0)	95 (76.6)		25 (75.8)	46 (83.6)		12 (80.0)	20 (80.0)	
	compressed	16 (20.0)	21 (16.9)		5 (15.2)	8 (14.5)		3 (20.0)	3 (12.0)	
	absent	8 (10.0)	8 (6.5)	.517§	3 (9.1)	1 (1.8)	.341*	0 (0.0)	2 (8.0)	.574*

[†]t-test; [*]Fisher Exact test; [§]Chi-squared test;

EDH: epidural hematoma, IPCH: intraparenchymal contusion or hematoma, LOS: length of hospital stay, PTCH: posttraumatic cerebral hydrocephalus, PTCI: posttraumatic cerebral infarction, SD: standard deviation.
